# A new aquareovirus causing high mortality in farmed Atlantic halibut fry in Norway

**DOI:** 10.1007/s00705-014-2235-8

**Published:** 2014-10-28

**Authors:** Steffen Blindheim, Are Nylund, Kuninori Watanabe, Heidrun Plarre, Børre Erstad, Stian Nylund

**Affiliations:** 1 Department of Biology, University of Bergen, 5020 Bergen, Norway; 2 Pharmaq Analytiq, Thormøhlensgate 55, 5008 Bergen, Norway

## Abstract

A new aquareovirus was isolated from cultured Atlantic halibut (*Hippoglossus hippoglossus*) fry at a facility where massive mortalities had occurred during the start-feeding phase. The same virus was also detected in juveniles (about 10 grams) of the 2013 generation at two other production sites, but not in larger fish from generations 2007–2012. The virus replicated in BF-2 and CHSE-214 cell cultures and produced syncytia and plaque-like cytopathic effects. This Atlantic halibut reovirus (AHRV) was associated with necrosis of the liver and pancreas, syncytium formation in these tissues, and distinct viroplasm areas within the syncytium in halibut fry. Transmission electron microscopy revealed that the viroplasm contained virions, non-enveloped, icosahedral particles approximately 70 nm in diameter with a double capsid layer, amorphous material, and tubular structures. The RNA-dependent RNA polymerase (RdRp) gene from the AHRV isolates showed the highest amino acid sequence identity (80 %) to an isolate belonging to the species *Aquareovirus A*, Atlantic salmon reovirus TS (ASRV-TS). A partial sequence from the putative fusion-associated small transmembrane (FAST) protein of AHRV was obtained, and this sequence showed the highest amino acid sequence identity (46.8 %) to Green River Chinook virus which is an unassigned member of the genus *Aquareovirus*, while a comparison with isolates belonging to the species *Aquareovirus A* showed <33 % identity. A proper assessment of the relationship of AHRV to all members of the genus *Aquareovirus*, however, is hampered by the absence of genetic data from members of several *Aquareovirus* species. AHRV is the first aquareovirus isolated from a marine coldwater fish species and the second reovirus detected in farmed fish in Norway. A similar disease of halibut fry, as described in this paper, has also been described in halibut production facilities in Canada and Scotland.

## Introduction

Atlantic halibut (*Hippoglossus hippoglossus*) production in Norway is limited to a few broodfish sites delivering fish to a small number of marine production sites. The dominating disease problems during the last 10 years have been related to Atlantic halibut nervous necrosis virus (AHNNV), infectious pancreatic necrosis virus (IPNV), and bacterial infections caused by *Aeromonas* sp and *Vibrio* spp. [1–5]. At some sites, mortality has been associated with liver necrosis with unknown aetiology [4]. Similar liver pathology has also been described in captive juvenile Atlantic halibut in Atlantic Canada [6]. The authors of that study concluded that the pathology was a result of an aquareovirus infection, and they found acute necrosis of proximal renal tubules in addition to the liver pathology. The fish were also infected with bacteria, but the causative agent of the disease and the observed mortality was believed to be of viral origin. A reovirus-like hepatitis in post-weaned farmed Atlantic halibut resulting in a mortality rate exceeding 95 % has also been described in Scotland [7]. However, none of these studies including sequencing of parts of the viral genome from the infected halibut, leaving the question about the virus identity open.

In 2013, we received material from a population of farmed Atlantic halibut that had experienced a high rate of mortality during weaning. The histopathology showed a resemblance to that described by Cusack et al. [6] and Ferguson et al. [7]. Transmission electron microscopy revealed the presence of virus-like particles with morphology similar to that of members of the family *Reoviridae*. Several members of this family have been isolated from fish, and the majority belong to the genus *Aquareovirus* [8, 9]. Aquareoviruses have been isolated from finfish and crustaceans and resemble the orthoreoviruses, but they possess 11 dsRNA genome segments and have a genome size of about 23 kbp. Aquareovirus particles are non-enveloped, spherical, multiple shelled, and about 80 nm in diameter. A typical cytopathic effect in cell culture is the production of large syncytia [10].

The aim of this study was to identify the virus associated with the high mortality of Atlantic halibut larvae at a hatchery in Norway, describe the histopathology in infected fish, and characterize the possible causative agent.

## Materials and methods

A high mortality rate was observed in juveniles of Atlantic halibut, *Hippoglossus hippoglossus*, that were kept in a commercial hatchery at 12 °C (80–90 % mortality), starting about 40 days after start-feeding (90–100 days after fertilization). The larvae were fed enriched *Artemia* but stopped eating prior to the onset of mortality. Moribund and dead larvae were removed daily from the affected tanks. Moribund larvae were collected from the rearing tanks and transported to the Fish Disease Research Laboratory at the University of Bergen in May 2013, where fish from three different tanks were processed for histology and histopathology (n = 14), and later transmission electron microscopy (n = 1). Five of the fish that were received, from two of the tanks experiencing mortality, were homogenized, sterile filtered (0.2 µm), and inoculated onto a selection of different cell cultures for virus isolation. Lastly, 10 larvae from the same two tanks were used for RNA extraction and downstream real-time RT-PCR. Water samples from the main sea water intake, and before and after the brood fish tanks, were filtered, RNA was extracted as described by Andersen et al. [11], and *Artemia* used as feed were sampled for real-time RT PCR analysis.

In addition to this first batch of fish, 79 larvae from both affected and unaffected tanks were stored at −80 °C until RNA extraction. Lastly, in order to examine whether the virus was present in older fish, 63 halibut of different generations (2007–2013, between 5 and 15 fish per generation) were collected from four different production sites in western Norway. Gill, liver, kidney and brain tissues from these individuals were stored at −80 °C until RNA extraction, and liver (n = 63) and kidney (n = 43) samples were used for real-time RT-PCR screening.

### Histology

The larvae were cut in two and fixed by immersion in a modified Karnovsky fixative in which the distilled water had been replaced by a Ringers solution. The fixative contained 4 % sucrose. Before embedding in EMBED-812 (Electron Microscopy Sciences), the tissues were stained/post-fixed in 2 % OsO_4_ for 60 minutes. Semi- and ultrathin sections were cut on a Reichert-Jung Ultracut E. The ultrathin sections (about 30 nm) were stained for 90 minutes in a 2 % aqueous uranyl acetate solution, followed by lead citrate. Semithin sections, 1.0 µm, were stained with toluidine blue. The semi- and ultrathin sections were used for examination of tissue changes and the possible presence of pathogens.

### Cell cultures

Histology and transmission electron microscopy (TEM) revealed the presence of reovirus- like particles in the liver and pancreas of the halibut larvae. The following cell cultures were tested as possible culture systems for these reovirus-like particles: BF2 cells (ATCC no. CCL-91), CHSE-214 cells (ATCC no. CRL-1681), RTgill cells, and ASK cells [12–15]. The cells were cultured in 75-cm^2^ tissue culture flasks at 20 °C in Eagle’s minimum essential medium (EMEM) supplemented with foetal bovine serum (10 %), L-glutamine (2 mM), non-essential amino acids (1 %), HEPES (10 mM) and gentamicin (20 μg ml^−1^). The cells were subcultured biweekly and formed monolayers within 2-7 days.

An aquareovirus, termed Atlantic halibut reovirus (AHRV), was isolated from the Atlantic halibut larvae collected from the two tanks with high mortality during start-feeding. For virus propagation, cell culture medium was removed from cell monolayers, cells were washed with PBS (pH 7.3), and sterile-filtered homogenate from the halibut larvae, diluted in serum-depleted medium (2 % FBS, L-glutamine, non-essential amino acids, HEPES, gentamicin), was added and allowed to adsorb for 90 minutes. The cells were incubated at 15 °C for 4–5 weeks, or until cytopathic effect (CPE) was observed. For all virus isolation procedures, a negative control of each cell culture type used was included and treated similarly to the infected cells.

### RNA extractions

The extraction of RNA from the halibut, artemia, and cell cultures were done as described by Devold et al. [12]. The RNA samples were stored at −20 °C.

### RT-PCR, sequencing and real-time RT-PCR

Primers targeting conserved areas in segments two and seven from chum salmon reovirus (accession nos. AF418295 and AF418300), Atlantic salmon reovirus (Accession nos. EF434978 and FJ652575) and a turbot aquareovirus (accession nos. HM989931 and HM989936) were constructed (Table 1). All PCR products were processed using ExoSAP-IT^®^ reagent (Affymetrix^®^) and sequenced using a BigDye Terminator Sequencing Kit (Applied Biosystems). Sequencing was done on PCR products, and all products were sequenced at least twice. Sequencing was performed at the sequencing facility at the University of Bergen (http://seqlab.uib.no/).

**Table 1:** Primers used to obtain the RNA-dependent RNA polymerase (RdRp) (accession no. KJ499467) and the fusion-associated small transmembrane (FAST) (accession no. KJ913664) protein genes from the AHRV isolates

Code	Primer	Position
RdRp		
AHRV-2F16	GTT TTA TCC ACT ATG TCC GC	0 - 20
ReoF1	TTG TTC AAC GCS CTR CCW C	22 - 40
AHRV-2R9	ACC AAC GAA TAT GTT AGA TGG	213 - 193
AHRV-2F12	GGA AGA TTA TTG GGA ACT GAA AG	222 - 244
AHRV-2F6	CGG AGT TCG TGC TTA CCT TG	787 - 805
AHRV-2R12	CCT TTT CGA TAC ATG CTG TG	896 - 877
AHRV-2R2	AGC CAA TGC CTG TTT GAG GG	1427 - 1446
AHRV-2F5	CCC TCA AAC AGG CAT TGG CTC	1427 - 1447
AHreoF10	GCT TCA ATC ACC TAC GCC TG	1804 - 1823
AHRV-2F13	GGA ATC CAG CAG CTC CTT AC	1925 - 1944
AHRV-2F15	CAG CTC CTT ACA ACC GAC CC	1934 - 1953
AHRV-2R6	TGG CGT GTT TCG GGA CGT AC	2167 - 2148
AHRV-2F2	TCA GAA CAA TTA CGT CTG CC	2208 - 2227
AHRV-2R13	ATA CTC GGC AGA GTC AGA GC	2376 - 2357
AHRV-2F7	CAC CCG AGA CAG GAG AGA TTT G	2453 - 2474
AHRV-2R15	GTG ACA TCG CAA ATG GTG TC	2685 - 2704
AHRV-2F10	AGC CCA AAC CTT CAG CCA TC	3122 - 3141
AHRV-2F11	GAC GAT CAT TGG TAC AAG ATC	3304 - 3324
AHRV-2F14	ACC AGC ACC ATC TCA TTG AC	3533 – 3542
AHRV-2R10	GCW GCA CTC ATY GCY TCC AC	2954-2935
AHRV-2R4	ATT AAA CCA CTA GCC GCC GC	3′ end
FAST		
AHRV-7F2	TCG AGC ACG GTC CAT CAT GC	1 – 20
AHRV-7R1	TGC TGG GTC ATG GTC TGC TC	377 – 358
AHRV-7F	TCG AGC ACG GTC CAT CAT	1-18
AHRV-7F1	GCC GCC AGT GTG ATG GAT ATC	52-72

The partial sequences of a segment believed to be the RNA-dependent RNA polymerase (RdRp) gene from two Atlantic halibut reovirus isolates, AHRV241013 and AHRV060513, have been deposited in the GenBank database under the accession numbers KJ499467 and KJ499468, respectively. The accession number for the partial sequence of a possible FAST protein gene from AHRV060513 is KJ913664.

Based on the partial sequence from the putative FAST protein gene (Segment 7) a TaqMan real-time RT-PCR assay was developed (Table 2). This assay was used in all testing of the RNA from halibut tissues and cell cultures inoculated with homogenates from the moribund halibut larvae. The RNA from the tissues of the halibut juveniles was also tested for the presence of Atlantic halibut nervous necrosis virus (AHNNV) and infectious pancreatic necrosis virus (IPNV) using TaqMan real-time RT-PCR assays [16, 17]. During the real-time RT-PCR screening, a housekeeping gene, elongation factor 1 alpha (EF1A1), was used as an internal control [18].

**Table 2:** Real-time RT-PCR assay targeting a gene segment coding for the putative fusion-associated small transmembrane (FAST) protein gene (accession no. KJ913664) from AHRV

Code	Probe/primer	Position
AHRV-7F	CCC GTA TTA GCA GTT ATC CTG TAT C	118 - 142
AHRV-7probe	GAT CCC ATG ATC GGT GAG G	168 - 186
AHRV-7R	CCC CAT CCT GCA CAT TCA AG	219 - 238

### Phylogeny

Sequence data were analyzed and assembled using VectorNTI software. The partial amino acid sequences of the putative RdRp and FAST proteins from AHRV were aligned with homologous protein sequences from selected reoviruses already available in the EMBL nucleotide database. To perform pairwise comparisons between the different virus proteins, the multiple sequence alignment editor GeneDoc (available at http://www.psc.edu/biomed/genedoc) was used. Polymorphic regions were aligned manually.

Phylogenetic analysis of the alignment of the putative RdRp was performed using the software TREE-PUZZLE 5.2 with maximum likelihood (ML) as optimality criterion, and the VT matrix for amino acid substitution. Bootstrapping of ML trees was done by 1000 quartet puzzling (QP) steps. Phylogenetic trees were drawn using TreeView [19].

## Results

The production site for halibut larvae had repeatedly been losing larvae during start-feeding and through the weaning phase for the last two years. The mortality rate ranged from 80 to 90 % in the different production tanks. During the period when mortality was observed, the larvae stopped eating, became passive, showing no escape behaviour, and died after a few days. The affected larvae were all positive for the presence of a member of the aquareoviruses (here named Atlantic halibut reovirus, AHRV), and negative for the presence of nervous necrosis virus (NNV) and infectious pancreas necrosis virus (IPNV). The Ct values obtained from this initial batch (n = 10), using the AHRV-7 real-time RT-PCR assay, ranged from 17.8 to 22.1. No parasites or bacteria were observed during the microscopic examination of the moribund larvae. The sea water intake and the artemia used as feed were negative for the presence of AHRV.

Of the 79 larvae received after the initial batch, 32 were from tanks with a high mortality rate (3 different tanks), and 31 of these were positive for the Atlantic halibut reovirus by real time RT-PCR screening (Ct values 18.6–28.9). Twenty-seven larvae came from a tank with halibut showing abnormal behaviour and were sampled twice, only a few days apart (n = 6 followed by n = 21). The Ct values obtained for this tank ranged from 20 to 37, with increasing prevalence from the first to the second sampling (50 % and 95 %, respectively). The last 20 larvae were from two tanks in which the larvae showed normal behaviour (n = 6 and n = 14) and were negative for the aquareovirus by real-time RT-PCR.

The tissue samples (liver, kidney) collected from different generations (2007–2013) of halibut at four different production sites in western Norway showed the presence of AHRV only in the 2013 generation at two production sites, with the exception of one positive in the 2010 generation at one of these sites. The positive fish from the 2013 generation had Ct values (AHRV-7) ranging from 20.0 to 32.0, while the housekeeping gene had Ct values around 16.0. Bacteria or parasites were not found in any of the tissues from these generations. However, the 2007-2012 generations from one site were positive for the presence of a microsporidian, possibly the one previously observed by Nilsen et al. [20].

### Histopathology

**Fig. 1:**
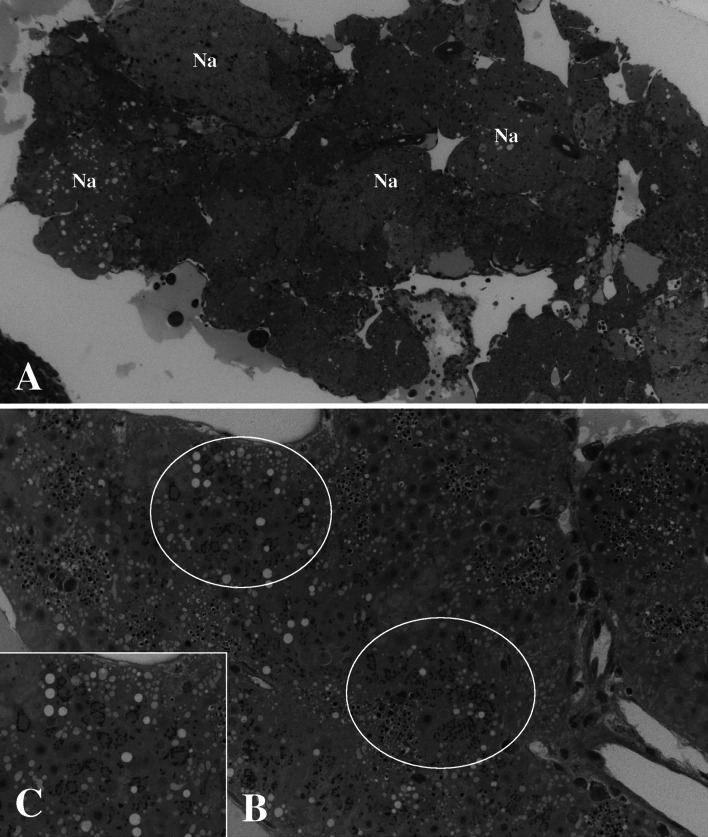
**A.** Multifocal necrosis (Na) in the liver of Atlantic halibut fry. **B**. Areas in pancreas tissue with formation of syncytia containing viroplasm (circles). **C**. Large subcellular inclusion, viroplasm (arrows), present in the pancreatic tissues

Sections of the moribund larvae showed multifocal necrosis in the liver and pancreas and areas of syncytial formation in these tissues (Fig. 1). Large subcellular inclusions were present in the syncytial areas (Fig. 1). There were no signs of inflammation in these areas. Food material could be observed in the gut of some individuals.

**Fig. 2:**
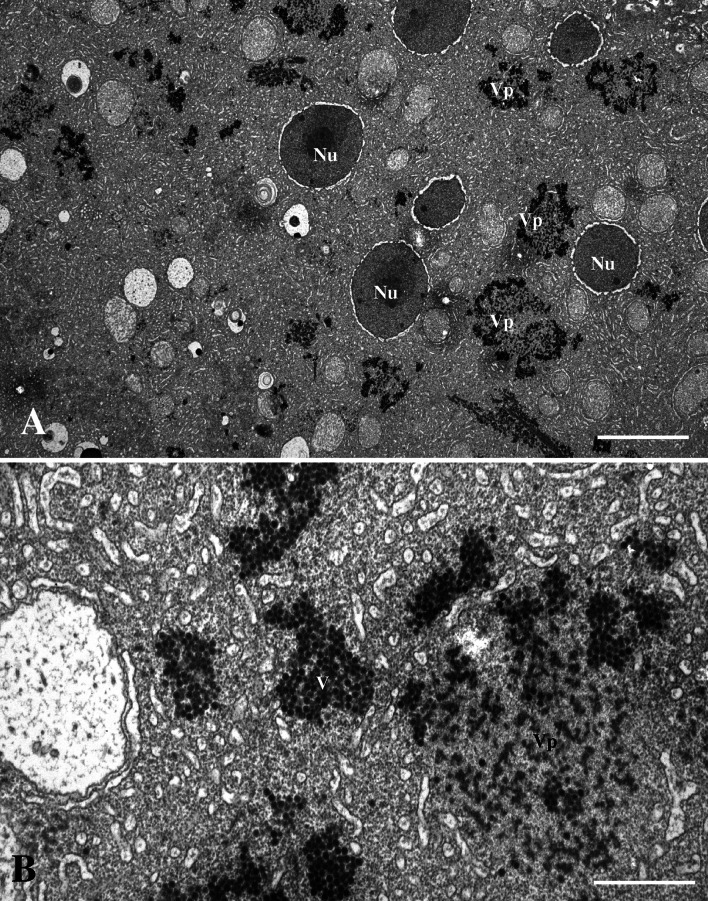
**A.** Viroplasm (Vp) within a syncytial area in the liver. Cell nucleus (Nu). Bar = 5.0 µm. **B.** The viroplasm (Vp) consists of amorphic material with variable electron density and contains virions (V). Bar = 1.0 µm

**Fig. 3:**
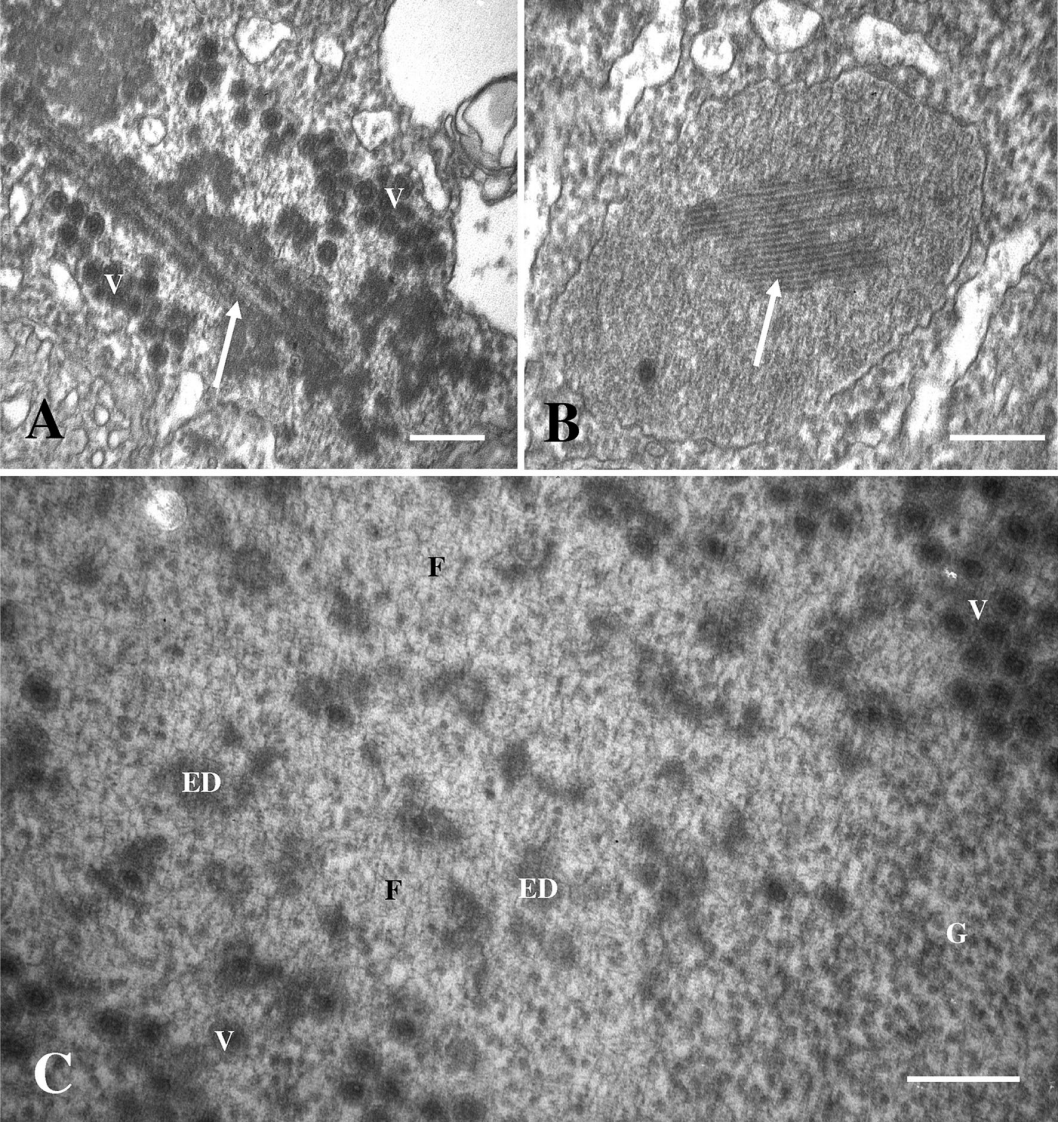
**A** and **B.** Tubular structures (arrow) in the viroplasm. Virions (V). **A.** Bar = 200 nm. **B.** Viroplasm with tubular inclusions (arrow). Bar = 200 nm. **C.** Magnification of the central area of the viroplasm containing granular material (G), fibre-like material (F), and small electron-dense areas (ED). Bar = 200 nm

**Fig. 4:**
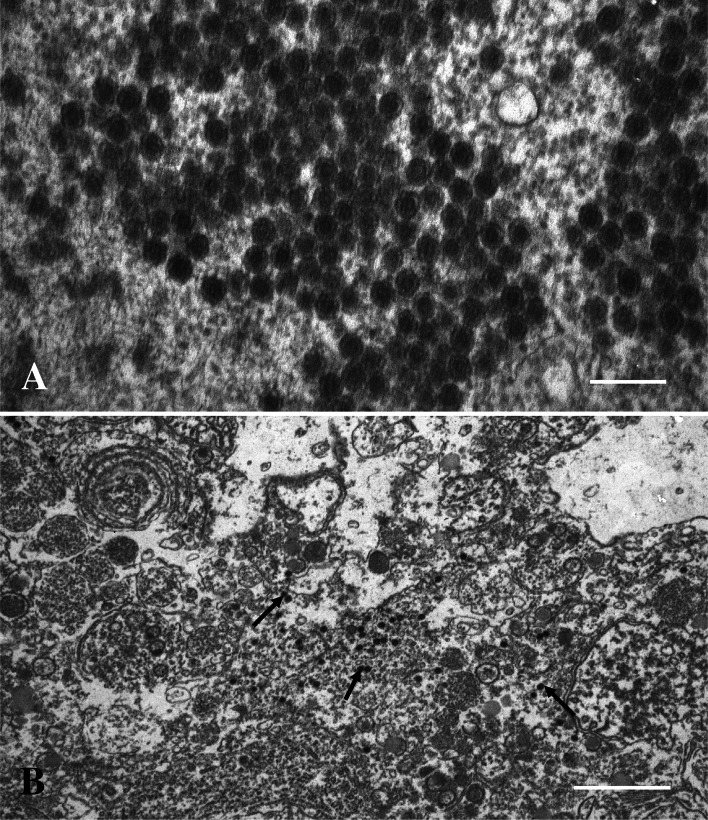
**A.** Mature virions of approximately 70 nm in diameter. Some of the virions show a hexagonal shape (icosahedral particles). Bar = 200 nm. **B.** Section from a necrotic part of the liver showing cell debris and virions (arrows). Bar = 1.0 µm

Transmission electron microscopy (TEM) showed that the subcellular inclusion in the syncytial areas in the liver and pancreas were viroplasm containing large amounts of viruses (Fig. 2A). The viroplasm, which was not surrounded by a membrane, consisted of amorphous material that constituted areas with variable electron density and virus-like particles (Fig. 2B). Tubular structures were also observed in the viroplasm, and at high magnification, the more electron-lucent areas of the viroplasm contained short, fibre-like structures (Fig. 3). The most frequently observed particles were spherical, about 70 nm in diameter, lacked an envelope, and had what seemed to be a double capsid structure (Fig. 4A). The inner capsid was about 34 nm in diameter. Smaller particles, about the same size as the inner capsid, were also observed in the viroplasm. These virus-like particles had a reovirus-like morphology.

The necrotic areas in liver and pancreas tissues lacked cellular structure and consisted of cellular debris and virions (Fig. 4B).

### Cell cultures

**Fig. 5:**
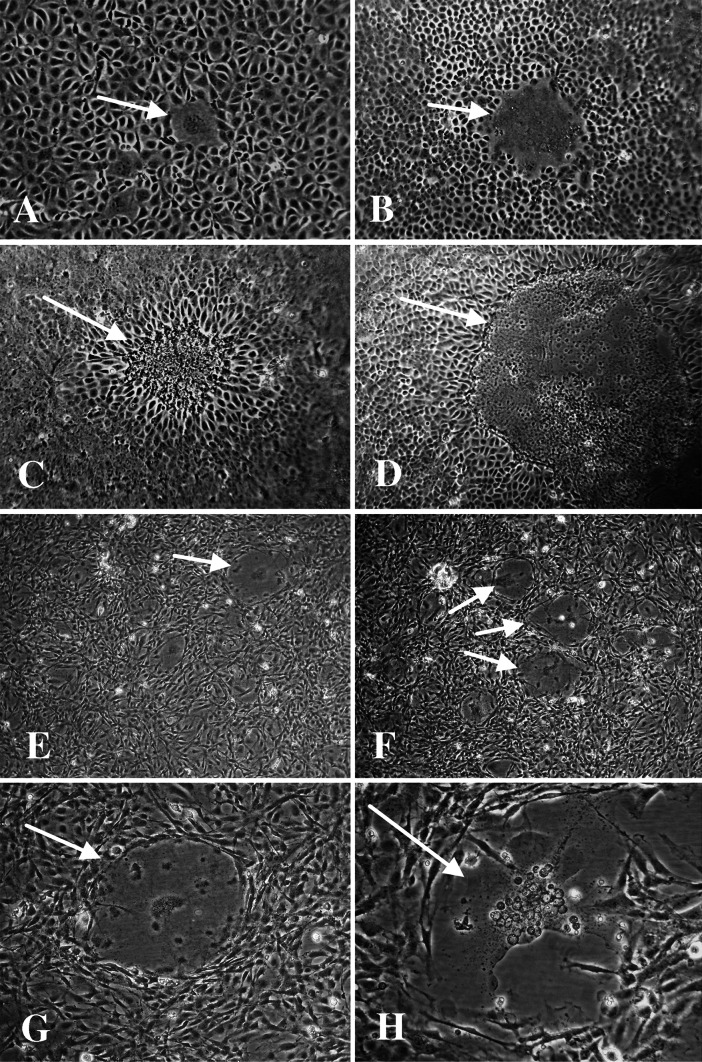
Cytopathic effect produced by Atlantic halibut reovirus (AHRV) in cell cultures. **A** **– D**. Different stages in the formation of a cytopathic syncytium in CHSE-214 cells infected with AHRV. **E-H**. Different stages in the formation of a cytopathic syncytium in a culture of BF-2 cells infected with AHRV

There was a progressive development of cytopathic effect (CPE) in both BF-2 cells and CHSE-214 cells. Microscopic examinations of the infected cell cultures showed multiple discrete plaques containing a central syncytium surrounded by slightly elongated cells at the margins of the syncytia (Fig. 5). The syncytium consisted of large, multinucleated cells, appeared after 6 days at 15 °C in BF-2 cells, and continued to grow until most of the monolayer was affected. The loss of these syncytial cells by lysis and the loss of the monolayer occurred after 11-13 days. This plaque formation progressed until most of the monolayer became affected. Subsequent passage of the supernatant from the infected cells did not result in CPE in BF-2 or CHSE-214 cells unless the supernatant was first subjected to freeze/thaw cycles (−80 °C and 20 °C). Testing of the supernatant and the remaining cells layer of the infected BF-2 cells before each passage resulted in Ct values as low as 12 using the real-time RT-PCR assay (AHRV-7 assay).

The development of CPE in CHSE-214 cells was similar to that observed in BF-2 cells, but at a slower rate, and the first syncytia were observed after 10 days (Fig. 5). The progression of CPE did not lead to a complete loss of the monolayer, but after 16 days, the loss of the syncytial cells started. Testing of the supernatant and the remaining cell layer in the infected CHSE-214 cells before each passage resulted in Ct values slightly above 20 (AHRV-7 assay).

No CPE was observed in ASK cells and RTgill cells, and the Ct values obtained using the AHRV-7 assay were always above 25 (range 25–30). The negative controls in all virus isolation procedures showed no CPE, and testing of cells from negative controls by real-time RT-PCR using the AHRV-7 assay gave negative results (data not shown).

### Partial genome sequence of AHRV

Using primers targeting the RdRp gene from aquareoviruses (accession nos. AF418295, EF434978, and HM989931), the nearly complete sequence (3624 and 3246 nucleotides) from a segment containing one large open reading frame (ORF) were obtained from the two aquareovirus isolates, AHRV241013 and AHRV060513 (accession nos. KJ499467 and KJ499468). This segment codes for a protein with an RdRp catalytic domain between amino acids 551 and 799 predicted using Motif Scan (http://myhits.isb-sib.ch/cgi-bin/motif_scan). The conserved polymerase amino acid motifs DXXXXD (motif A), SG (motif B) and GDD (motif C), present in all members of the genera *Aquareovirus* and *Orthoreovirus*, were identified at positions 592–597, 649–650 and 740–742, respectively (accession no. KJ499467). Alignment of the nucleotide and the predicted amino acid sequences of AHRV with RdRp gene sequences from other aquareoviruses showed the highest similarity to chum salmon reovirus (CSRV-CS) and Atlantic salmon reovirus (ASRV-TS), respectively. Both isolates belong to the species *Aquareovirus A* (Table 3). The amino acid sequence identity between the putative RdRp from AHRV and turbot reovirus (SMReV) was 76.7 %, while the amino acid similarities to other aquareoviruses sequenced were below 60 %.

**Table 3:** Comparison of the nucleotide (3624 nt) and predicted amino acid (1204) sequences from AHRV (accession no. KJ499467) with selected members of the genus *Aquareovirus* and piscine reovirus (PRV)

Virus	Year	nt %	aa %	Accession no.	Host
Aquareovirus A					
CSRV-CS	1981	69.9	79.3	AF418295	*Oncorhynchus keta*
ASRV-TS	2007	69.3	80.1	EF434978	*Salmo salar*, Tasmania
Aquareovirus C					
GSRV	1977	58.5	59.1	AF403399	*Notemigonus crysoleucas*
Aquareovirus G					
AGCRV PB01-155	2004	58.4	58.0	EF589099	*Ctenopharyngodon idella*
Unassigned					
SMReV	2007	69.6	76.7	HM989931	*Scophthalmus maximus*
GCRV GD108	1990	54.2	43.9	HQ231199	*Ctenopharyngodon idella*
GCRV 104	2009	54.1	41.9	JN967630	*Ctenopharyngodon idella*
PRV GP2010/NOR	2010	38.5	52.9	GU994015	*Salmo salar, Norway*

A partial sequence was also obtained from a segment that might code for a fusion-associated small transmembrane (FAST) protein (accession no. KJ913664). This sequence showed the highest nucleotide and predicted amino acid sequence similarity (52.6 % and 46.8 %) to Green River Chinook virus (accession no. KC588381), which is an unassigned member of the genus *Aquareovirus*, while the amino acid sequence identity compared to members of the species *Aquareovirus A* is <33.0 % (Table 4). The real-time RT-PCR assay (AHRV-7) used in this study targets this gene sequence from the two AHRV isolates.

**Table 4:** Comparison of the partial amino acid (126) sequence from a putative FAST protein from AHRV (accession no. KJ913664) with the corresponding sequence from selected members of the genus *Aquareovirus*

Virus	Year	aa %	Accession no.	Host
Aquareovirus A				
CSRV-CS	1981	27.0	AF418300	*Oncorhynchus keta*
ASRV-Canada-2009	2009	32.5	ACN38056	*Salmo salar*, Canada
Aquareovirus C				
GSRV	1977	19.8	NP_938067	*Notemigonus crysoleucas*
GCHR 873	-	19.0	AAF91306	*Ctenopharyngodon idella*
Aquareovirus G				
AGCRV PB01-155	2004	19.8	YP_001837101	*Ctenopharyngodon idella*
Unassigned				
SMReV	2007	31.7	ADZ31983	*Scophthalmus maximus*
Green River Chinook virus	-	46.8	AHJ14806	*Oncorhynchus tshawytscha*
Channel catfish reovirus 730	-	19.0	ADP05120	*Ictalurus punctatus*

### Phylogeny

**Fig. 6:**
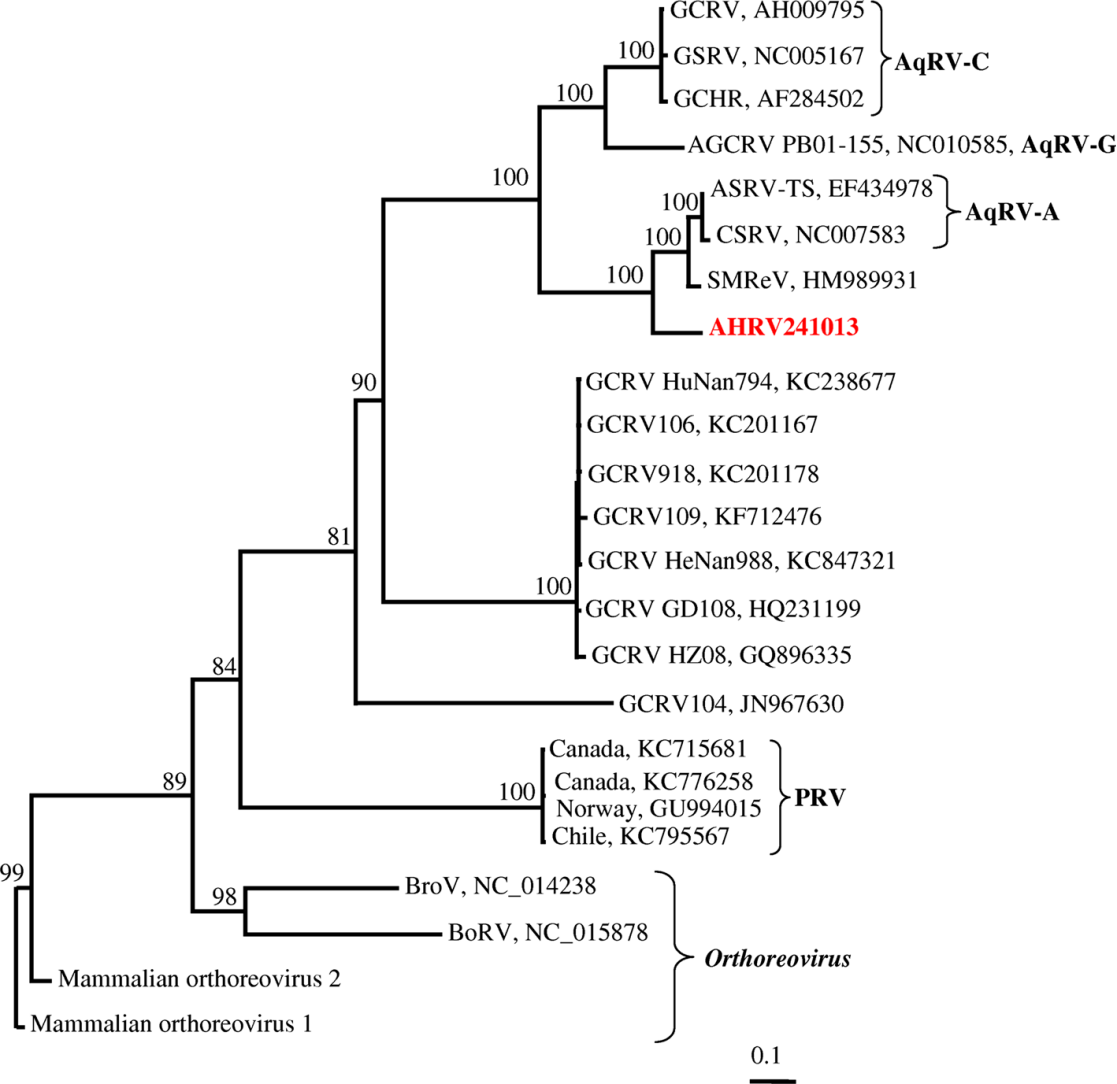
The phylogenetic position of the AHR virus based on analysis of the RNA-dependent RNA polymerase from selected members of the genera *Aquareovirus* and *Orthoreovirus*. Four orthoreoviruses (baboon orthoreovirus, BoRV; Broome virus, BroV; mammalian orthoreovirus 1; and mammalian orthoreovirus 2) were used as outgroups. The support values are frequencies (%) at which a given branch appeared in 1000 bootstrap replications

A phylogenetic tree showing the relationship of AHRV to other members of the genus *Aquareovirus* was constructed based on the available RdRp sequences (Fig. 6). AHRV clusters close to a clade containing CSRV, ASRV-AS and the turbot virus SMReV. American grass carp reovirus (AGCRV; species *Aquareovirus G*), is the second closest relative, while the other known Norwegian fish reovirus, piscine reovirus (PRV), is only distantly related to AHRV.

## Discussion

An aquareovirus was isolated from Atlantic halibut larvae from a facility with a high mortality rate during the start-feeding phase (Table 3). This is the second member of the genus *Aquareovirus* isolated from strictly marine fish [21]. The larvae did not show any clinical signs of infection with parasites or bacteria. The observed mortality rate and results of histopathology and TEM studies showed changes similar to those described in moribund Atlantic halibut larvae in Canada and Scotland [6, 7]. The viroplasm and the morphology of the virions in the syncytial areas are also similar to those described in the two published studies. However, we did not find any changes in renal tissues as described by Cusak et al. [6] or syncytial giant cell formation of the mucosal epithelium of the intestine [7], but we did find severe lesions, with syncytial formation and necrosis, in the liver and pancreatic tissues. Mild pancreatitis, with influx of neutrophils and macrophages, was seen in some of the specimens from Scotland [7], while such inflammatory responses were not seen in the pancreatic tissues of the larvae in the present study. Despite some slight differences the histopathology, the disease appears to be same as that described in the two earlier studies, and the reovirus-like virions match the description given in the two papers [6, 7].

Species demarcation criteria for the genus *Aquareovirus* have traditionally been based on number of genome segments, host range and disease symptoms, number of capsid layers, presence of spiked or unspiked cores, cross-hybridization, electropherotype analysis by gel electrophoresis, serological comparison, ability to reassort during mixed infections, conserved terminal sequences, and RNA and amino acid sequence analysis [10]. However, members of a species in the genus *Aquareovirus* may also be identified based on the identity of RdRp sequences. Members of the same species should have >95 % amino acid (aa) sequence identity, while the corresponding values between species are 57–74 % [10]. Based on amino acid sequence identity of the two AHRV isolates, with the highest identity (80 %) to a member of the species *Aquareovirus A* (ASRV-TS), this virus is put in an uncertain position, with less than 95 % aa identity but higher than 74 % aa identity.

The host range of members assigned to the species *Aquareovirus A* includes American oyster and anadromous or fresh water fish only, while the host for AHRV is a strictly marine fish, the Atlantic halibut (*Hippoglossus hippoglossus*) [22–25]. The closest aquareovirus marine relative of AHRV, based on the identity of the RdRp gene (SMReV, 76.7 %), has been isolated from turbot (*Scophthalmus maximus*) in China [21]. Turbot is a warm (temperate)-water species, while halibut is a cold-water species with little sharing of habitats with turbots. Hence, the host species, halibut, suggests that AHRV could be a member of a new distinct species within the genus *Aquareovirus*. However, more data are needed before a clear conclusion about the taxonomical status of the AHRV can be reached. A major problem for a proper assessment of the relationship of AHRV with respect to all aquareoviruses is the absence of genetic data from members of several species and unassigned reoviruses that could be members of the genus *Aquareovirus*.

The cytopathic effect (CPE) in the two cell cultures, BF-2 cells and CHSE-214 cells, showed discrete plaques containing large syncytia and elongated cells around the plaque margins. This CPE is characteristic for many *Aquareovirus* but does also occur with many orthoreoviruses [26, 27]. The fusion-associated small transmembrane (FAST) proteins are responsible for the induction of syncytia and may also trigger apoptosis [28–30]. The ability of the AHRV to cause syncytia in cell cultures suggests that this virus also carries a FAST protein. This is also supported by the partial sequences obtained from the AHRV isolates, showing 46.8 % nucleotide sequence identity to the FAST protein genes from the unassigned aquareovirus Green River Chinook virus. The identity compared to the recognized member of the species *Aquareovirus A* (CSRV) was <33.0 %.

AHRV is the second member of the family *Reoviridae* detected in farmed fish in Norway, the first being the unassigned virus, piscine reovirus (PRV), associated with heart and skeletal muscle inflammation in Atlantic salmon [31]. PRV is widespread in both farmed and wild Atlantic salmon in Norway [32], while AHRV has only been detected in farmed halibut fry. Future studies will show if this virus is also associated with the halibut fry mortalities in Canada and Scotland, and if AHRV is present in wild halibut in Norway.
